# Object recognition is enabled by an experience-dependent appraisal of visual features in the brain’s value system

**DOI:** 10.1016/j.neuroimage.2020.117143

**Published:** 2020-11-01

**Authors:** Vladimir V. Kozunov, Timothy O. West, Anastasia Y. Nikolaeva, Tatiana A. Stroganova, Karl J. Friston

**Affiliations:** a MEG Centre, Moscow State University of Psychology and Education, Moscow, 29 Sretenka, Russia; b Nuffield Department of Clinical Neurosciences, John Radcliffe Hospital, University of Oxford, Oxford, OX3 9DU, UK; c Wellcome Trust Centre for Neuroimaging, 12 Queen Square, University College London, London, WC1N 3AR, UK

**Keywords:** Visual perception, Object recognition, Mooney figure disambiguation, Magnetoencephalography, Value system, Prior experience, Predictive coding, Region-based multivariate pattern analysis, Representational similarity analysis, Temporal cross-decoding generalization

## Abstract

This paper addresses perceptual synthesis by comparing responses evoked by visual stimuli before and after they are recognized, depending on prior exposure. Using magnetoencephalography, we analyzed distributed patterns of neuronal activity – evoked by Mooney figures – before and after they were recognized as meaningful objects. Recognition induced changes were first seen at 100–120 ​ms, for both faces and tools. These early effects – in right inferior and middle occipital regions – were characterized by an increase in power in the absence of any changes in spatial patterns of activity. Within a later 210–230 ​ms window, a quite different type of recognition effect appeared. Regions of the brain’s value system (insula, entorhinal cortex and cingulate of the right hemisphere for faces and right orbitofrontal cortex for tools) evinced a reorganization of their neuronal activity without an overall power increase in the region. Finally, we found that during the perception of disambiguated face stimuli, a face-specific response in the right fusiform gyrus emerged at 240–290 ​ms, with a much greater latency than the well-known N170m component, and, crucially, followed the recognition effect in the value system regions. These results can clarify one of the most intriguing issues of perceptual synthesis, namely, how a limited set of high-level predictions, which is required to reduce the uncertainty when resolving the ill-posed inverse problem of perception, can be available before category-specific processing in visual cortex. We suggest that a subset of local spatial features serves as partial cues for a fast re-activation of object-specific appraisal by the value system. The ensuing top-down feedback from value system to visual cortex, in particular, the fusiform gyrus enables high levels of processing to form category-specific predictions. This descending influence of the value system was more prominent for faces than for tools, the fact that reflects different dependence of these categories on value-related information.

## Highlights

• Recognition effects following Mooney figure disambiguation were decoded from MEG data.
• Neural changes occur in 3 stages that challenge the hierarchical view of perception.
• Category-specific activity in the brain’s value system precedes that in visual cortex.
• Face-specific response in the right fusiform gyrus is delayed for disambiguated faces.
• The top-down influence of the value system is more prominent for faces than for tools.

## 1 Introduction

A core assumption of cognitive neuroscience is that visual perception involves hierarchically organized processing, the outcomes of which span from low-level visual features to increasingly abstract and subjective representations. One of the most intriguing aspects of this process is the attribution of meaning to stimulus that only exists in reference to the state of the observer. Accordingly, it has been suggested that perception is not only determined by the bottom-up propagation of information from our sensory organs, but is also depends upon the top-down influence of prior knowledge in cortical hierarchies (Oliva and Torralba, 2007; Rauss et al., 2011; Gilbert and Li, 2013; Piëch et al., 2013; Summerfield and De Lange, 2014; de Lange et al., 2018). This experimental study was motivated by the question: how does prior knowledge affect visual processing to facilitate object recognition?

Top-down influences in the brain are an integral part of predictive processing (Rao and Ballard, 1999; Friston, 2005), in which higher levels of the cortex generate knowledge-based predictions about representations at lower levels. When predictions are incomplete or incompatible with representations at the lower area, a prediction error arises – that is thought to update representations at higher levels. The ensuing predictive coding architecture suggests that perception relies upon the combination of signals encoding either predictions or prediction errors. The signals are thought to be processed in two distinct neural populations and propagate in opposite (top-down vs bottom-up) directions. This characteristic of the prediction coding framework can explain why previous experimental evidence – investigating the role of prior knowledge in perceptual processing – has often yielded conflicting, inconsistent, or controversial results. Under the predictive coding architecture, it would be expected that recognition would elicit distinct effects in the two subpopulations: enhancement of neuronal responses in units generating predictions, and a concomitant suppression of prediction error units (Mumford, 1992; Friston, 2005). The assumption that the corresponding neural subpopulations co-exist within the same (coarse grained) cortical region was confirmed by a recent study (De Gardelle et al., 2013) and could explain why recognition facilitation experiments – in studies with low spatial resolution – evince response suppression or enhancement, depending on the specific experimental conditions (Segaert et al., 2013) or the exact level of the brain region investigated within the perceptual hierarchy (Murray et al., 2002).

Another issue that complicates the interpretation of neurophysiological evidence for predictive coding is that perceptual inference depends on prior experience that precedes the recognition of objects of a particular category. For categories such as faces, experimental results indicating neurophysiological correlates of predictions have been found, whilst for other categories (such as houses for instance), no such correlates have been found (Summerfield et al., 2006; Trapp et al., 2016; Brodski-Guerniero et al., 2017). Recent studies (Mahon et al., 2009; Bi et al., 2016; Peelen and Downing, 2017) have reported that the category of an object interacts with the ability to rely on visual input: selectivity for artifacts and scenes is largely immune to visual deprivation, whereas selectivity for animate items is found only in sighted individuals, when processing visual stimuli. Moreover, it has been shown that the perception of faces is accompanied by a repetition-enhancement effect, with an increase in activity distinguishing faces from nonsense images (predominance of prediction-based effect), while manipulable artifacts (e.g., tools) typically elicit a repetition suppression of category specific responses (prevalence of error-based effect) (Kozunov et al., 2018).

In addition to the issues of interpretation due to differences in experimental design, there is a conceptual problem with the straightforward application of the predictive coding framework to visual perception. Predictions serve as constraints to reduce the uncertainty when resolving the ill-posed inverse problem of perception (Pizlo, 2001; Friston, 2003). To facilitate meaningful object recognition, a limited set of high-level, semantic predictions must be available before it can organize lower-level processing. However, in order for a percept to arise during free observation (in the absence of top-down constraints), the only way that semantic predictions could be informative is if they have been pre-selected on the basis of available visual cues. This suggests a fast bottom-up flow of stimulus-bound information that triggers a selection of high-level predictions, which then contextualize a slower predictive coding stream that processes more precise information.

Indeed, the fast processing of low spatial frequencies – in the magnocellular visual stream – has been suggested as a way to nuance predictive coding; providing a higher level ‘gist’ that contextualizes lower-level predictive coding – based upon high spatial frequency information in the parvocellular stream (Bar et al., 2006). This kind of perceptual processing was initially proposed to explain why global properties of a visual object are processed first (Navon, 1977; Badcock et al., 1990; Han et al., 2002). However, global (i.e. large-scale) configurations are still spatial in nature therefore they cannot inform predictive processing to form view-invariant representations (Quiroga et al., 2005). Such view-invariant representations have been shown to evolve from shape-dependent representations in the ventral occipito-temporal regions – along a posterior-to-anterior gradient – and follow a transition from low-level pixel-based visual features to the representation of shape (Bracci and Op de Beeck, 2016; Kaiser et al., 2016; Proklova et al., 2016). From Gestaltist principles of perception, it is claimed that even the perception of shape requires holistic (configural) properties that arise from interrelations between the individual parts – and thus cannot be derived from low-level visual features (Kimchi, 2015). Since the time of Gestaltists, additional principles have come into play (for a review, see Wagemans et al., 2012) and recently the domain of their application has been extended to configurations in semantic space (Pinna, 2010; Pinna et al., 2018). In this setting, a new figure-ground segregation principle was proposed to exert an influence of subjective meaning on the processing of visual patterns. Accordingly, it has been shown, that semantic memory can be accessed before an object is segregated from its background (Peterson and Gibson, 1994; Sanguinetti et al., 2014). This suggests that holistic configurations in spatial and semantic spaces can be coupled in order to influence object recognition in a general way and at an early stage of processing.

The principal difference between Gestalt and meaning-based configural principles is that the former are believed to be innate, whilst the latter depend on the previous experience. While the acquisition of new semantic categories may require long training procedures (Gauthier and Tarr, 1997; Seger and Miller, 2010; Brants et al., 2016), learning to recognize a particular visual pattern – as a member of a familiar category – can occur very quickly. In this study, we investigate changes of neural activity underlying a sudden disambiguation of Mooney figures (Mooney and Ferguson, 1951), which can be associated with the salience of the ‘‘Aha!’’ moment (Ludmer et al., 2011). Itti and co-authors (Itti et al., 1998) have suggested a model in which multiscale image features are combined into a single topographical *saliency map* used for rapid selection (in order of decreasing salience) of conspicuous locations that guide spatial attention. Since attention and perceptual grouping are closely related (Driver et al., 2001; Roelfsema, 2006; Desimone and Duncan, 1995), a model for salience-based object recognition – which incorporates spreading of attention across Gestalt cues (Wannig et al., 2011) – was recently proposed (Yu et al., 2016). However, in both of the above models, and as is the case in most of the literature, the term ‘salience’ is considered as a property of an image itself and thereby inextricably linked to exogenous spatial attention and so that ‘spreading’ of attention is limited to structures in geometric space.

At the same time, the idea of a saliency map has been extended to incorporate top-down signals that are related to behavioral goals (Corbetta and Shulman, 2002; Ipata et al., 2006; Shomstein, 2012). Moreover, some authors use the term ‘salience’ to designate a stimulus related characteristic that can be updated by an observer’s past experience (Gottlieb et al., 1998; Soto et al., 2005) – and includes nonspatial components like planning, estimation of elapsed time, and reward-based decisions (Balan and Gottlieb, 2009; Peck et al., 2009). Finally, the terms ‘emotional salience’ or ‘value-based salience’ are used to refer to endogenous salience which differs significantly from goal-based attention (Phan et al., 2004; Pauli and Röder, 2008; Niu et al., 2012). Edelman (2003) defines values as phenotypic characteristics of an organism that were selected during evolution and constrain somatic selective events. The structures comprising the limbic and paralimbic systems are involved in value processing. It has been shown that value-based salience is encoded whenever it is relevant or not relevant for the ongoing task (Lebreton et al., 2009) and can interact – and even reverse the effects of – sensory salience (Niu et al., 2012; Schütz et al., 2012; Pauli and Röder, 2008). One could conclude that a saliency map describes an interface between spatial attention (both exogenous and endogenous) and value-based selection (Compton, 2003).

Adolphs (2002) has introduced a two phase model for the appraisal of value-based aspects of a stimulus: (1) A subcortical path terminates in the amygdala and is specialized for very fast, automatic extraction of *primary inducers*; namely, characteristics of stimuli that convey intrinsic, biologically relevant values (Davis and Whalen, 2001; Bechara et al., 2006). This pathway in turn invokes a downstream modulation of sensory processing in the visual association cortices and leads to the emergence of complex features (e.g., shapes) that depend on configural properties. (2) Complex features feed into a network of frontal regions, primarily orbitofrontal cortex and insula, to recognize *secondary inducers* – characteristics invoking personal salience and conveying values based on the extent of personal associations learnt over past experiences (Lane et al., 1997; Anderson et al., 2003). Spreading of the secondary inducers provides experience-dependent feedback to fusiform and superior temporal cortex. This model suggests that in the same manner as fast magnocellular activity is in a position to modulate via feedback later parvocellular driven activity in the visual cortex, a similar feedback influence could be assigned to the value system.

We hypothesized that a learning to recognize of Mooney figures will result in familiarity-dependent change in a value-based saliency map. Familiarity is considered here in the dual-processes memory theory sense (e.g. Norman and O’Reilly, 2003) as a sign of ‘global match’ of the configuration to that encountered on previous occasions. It should be distinguished from both a priming; namely, a short-lasting effect caused by a repetition of the same stimulus that preceded it; as well as from repetition effects that admit presentations of intervening images in a row of repetitions of the same stimulus. There is no generally accepted or clear demarcation between these constructs and they are sometimes used interchangeably in the literature. In particular, there are conflicting opinions about whether priming and repetition effects represent distinct mechanisms (Bentin and Moscovitch, 1988; Ratcliff et al., 1985) or differ only quantitatively (Henson et al., 2004). In any case, it has been shown that repetition effects can be approximated by an exponential law and thereby attenuate after just a few repetitions (Stefanics et al., 2018). In contrast, familiarity invokes a kind of long-term memory, in which recognizability can be maintained over years. At the same time, the familiarity of a particular configuration can be acquired very quickly, after a single exposure (Brown and Aggleton, 2001; Diana et al., 2007; Gimbel et al., 2017). During perception, a reintegration of the combined spatial and value-space familiar configuration based on partial cues can provide top-down constraints on hierarchical predictive coding to optimize perceptual inference and endow representations with a value-based meaning or semantics. This contextualization of predictive processing can be articulated in terms of a change in salience induced by recognizing that the current visual input can be explained in terms of a familiar object.

In the study, we used magnetoencephalography (MEG) to investigate spatiotemporal patterns of neural activity underlying the recognition of Mooney figures. We manipulated the probability of recognition using prior exposure to stimuli. To do this, we used images that were specially processed, so that when seeing them for the first time, an observer only perceives a meaningless pattern of shapes but after a short period of training he/she can recognize a meaningful object in the image. This experimental design provides an attractive paradigm to study experience-dependent perceptual processes that are equipped with a top-down bias. This is because when presenting the same image before and after training, the sensory input is exactly the same, but the prior beliefs accumulated by the observer are fundamentally different. With respect to the type of experience underlying a particular category formation, we probed two categories: faces – an example of objects highly dependent on value-related information, and tools – a class of objects that are determined by what it was made for.

On the above view, experience dependent changes enable the brain to update saliency maps by changing the way it processes visual features and so provides evidence for high-level predictions. We expected that changes in the regions associated with value system would take a form of reorganization - change in the spatial structure without an overall power increase (decrease) of neural activity in the region. In order to investigate emergence of new patterns of activity corresponding to the reorganization of saliency maps we applied a multivariate decoding techniques: a region-based multivariate pattern classification analysis (RB-MVPA, Kozunov et al., 2018) followed by representational similarity analysis (RSA, Kriegeskorte et al., 2008) and a temporal cross-category generalization analysis (King and Dehaene, 2014; Kaiser et al., 2016). The basic idea behind this decoding tactic is to compare experimental conditions in a neural activity feature space of an individual subject and conceptualize differences in multiple vertices as a single value. Only following the transition to this scalar indicator of between-condition differences in brain activity we require similarity between subjects. The absence of the requirement of vertex-to-vertex consistency between different subjects, while keeping the ability to extract changes in an internal structure of brain region activity makes the decoding techniques appropriate for investigation of saliency maps alterations. This allowed us to test the hypothesis that during the perception of disambiguated stimuli effects of the reorganization of saliency maps should precede the emergence of category-specific patterns of activity in the high-level visual cortex.

## 2 Methods

### 2.1 Participants

Thirty-four volunteers (15 males, 19 females) with an average age of 24.6 years (SD ​= ​4.31) participated in the main experiment. This study was conducted in accordance with the recommendations of the Declaration of Helsinki with written informed consent from all subjects. The protocol was approved by the ethics committee of Moscow State University of Psychology and Education.

### 2.2 Stimuli

During the main experiment, we used 26 bitonal (black and white) Mooney images. To produce these images, we blurred ~100 grayscale photographs of faces, animals, plants, and tools with a Gaussian filter and binarized them using a custom routine written in MATLAB (MathWorks, Inc.). Some nonsensical fragments of real grayscale photographs were also subjected to the same procedure. All resulting images were 500 ​× ​500 pixels in size and equalized in luminance (number of white pixels) and length of the contours.

**Fig. 1:**
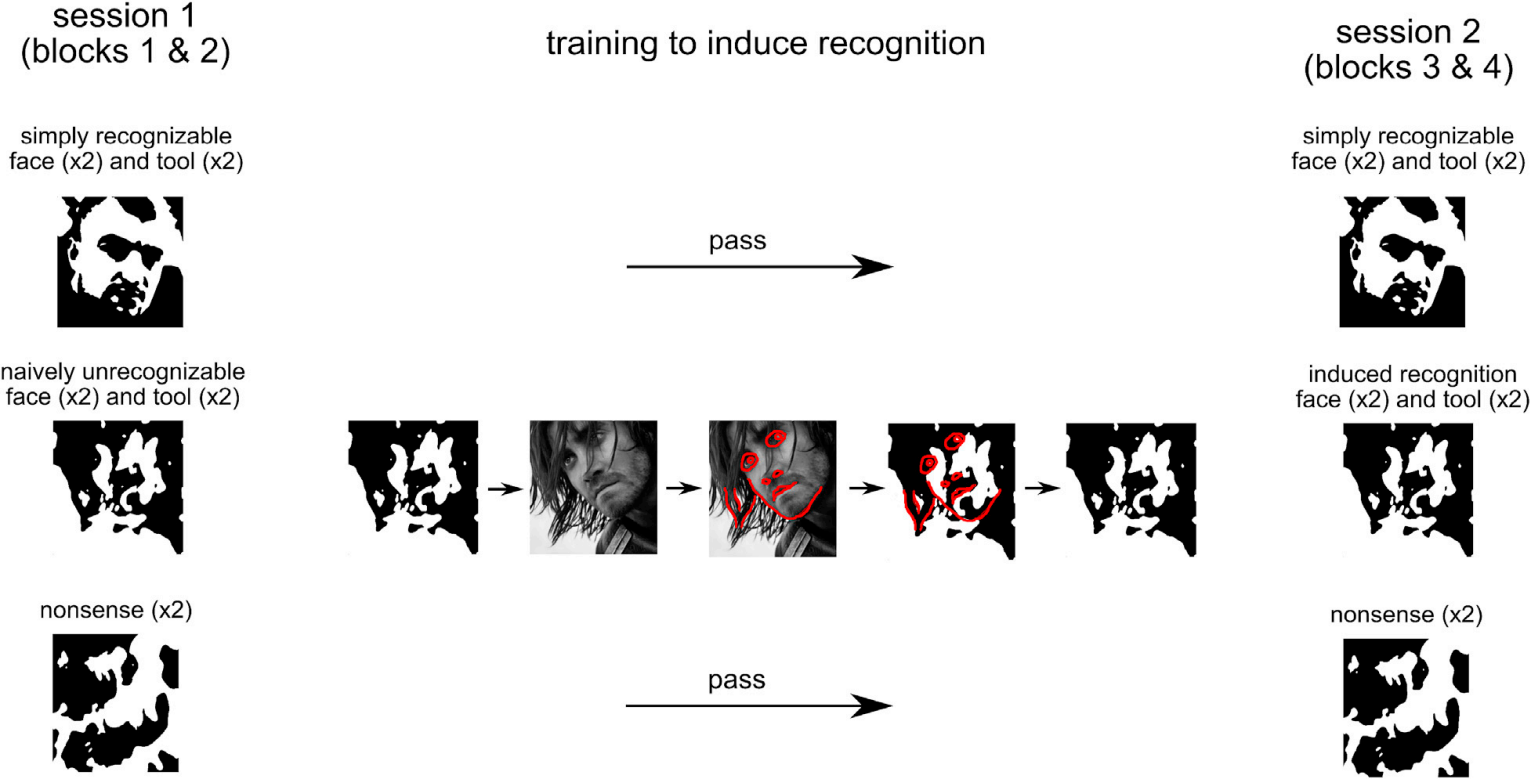
Summary of Experimental Design. We investigated the neuronal processing related to the perception of five classes of images, each comprising two samples (a total of ten images): *simply recognizable* faces, *simply recognizable* tools, *naively unrecognizable* faces, *naively unrecognizable* tools, and *nonsense* images. Additionally, an auxiliary set of bitonal images were presented to ensure a variety of stimuli were presented across the whole sequence (not shown here). The images were presented in two separate sessions, each comprising two blocks of presentations of image sequences. Between the two sessions we carried out a fast training procedure to induce recognition of the *naively unrecognizable* class of stimuli. The more detailed description of training procedure is given in section 2.3 of the Methods.

Following this, we formed the main subset of 10 stimuli that comprised: 2 *simply recognizable* faces, 2 *simply recognizable* tools, 2 *naively unrecognizable* faces, 2 *naively unrecognizable* tools, and 2 *nonsense* images. To assign stimuli to these different classes, a bank of images was shown to a group of 60 neurotypical volunteers, none of whom participated in the main experiment. *Simply recognizable* images were chosen if they were correctly identified by more than 95% of subjects, when seen for the first time. *Naively unrecognizable* images of faces and tools were selected, if they matched the following characteristics: they should be identified as meaningless by more than 90% of subjects, when seen for the first time but should be correctly identified as an object by more than 90% of subjects when seen after performing a procedure designed to induce stimulus recognition (detailed below and in Fig. 1). *Nonsense* images were chosen if they had been identified as meaningless by more than 95% of participants.

The remaining 16 stimuli constituted an auxiliary subset of images and comprised: 6 simply recognizable images of animals, 6 simply recognizable images of plants and 4 nonsense images. This subset of images was used to ensure that a variety of stimuli categories and exemplars were presented in the sequence.

### 2.3 Procedure

In the main experiment – for MEG data acquisition – the stimuli were displayed using Presentation software (Neurobehavioral Systems Inc., United States) and were back-projected onto a translucent white projection screen located 1.7m in front of the participants with a Panasonic PT-D7700E-K DLP projector to provide an 8 ​× ​8° visual angle. The images were presented with 1280х1024 screen resolution and 60 ​Hz refresh rate.

The stimuli were displayed within four 16-min blocks, separated by breaks of roughly 5 ​min each. All images from the main subset and four different images from the auxiliary subset were shown during each block. Every image from the main subset was presented 40 times during each block. Images from the auxiliary subset were displayed 15 times during each block. Evoked responses for the images from auxiliary subset were not analyzed. Stimuli were presented in a random order for duration of 800 ​ms. Randomization of the stimulus sequence was performed by simple shuffling of the rows of the scenario’s template (as it is implemented by Presentation’s ‘randomize’ option). Interstimulus interval (ISI: interval between the offset of one stimulus to the onset of another) took a random value from the interval 1000–1500 ​ms.

After each stimulus presentation, participants were required to name aloud with a single word (to be sure that vocalization ends before the next stimulus) what they had seen in the picture. We did not provide instructions about how to name any picture, except in the case of the nonsensical images; in which case we asked the participant to say “nothing” when they were unable to recognize a meaningful object. All subject’s responses were recorded by the experimenter and on a dictaphone. Later, during an offline analysis, categories were ascribed to each of the participant’s responses. We treated all appropriate responses as correctly categorized (for example, “woman” in response to the presentation of a woman’s face was treated as a correctly recognized face category). Only trials with correctly categorized stimuli were analyzed further.

Between the second and the third blocks, we carried out a fast training procedure to induce recognition of the *naively unrecognizable* type of stimuli. This training was not performed for the *simply recognizable* or *nonsense* classes. The procedure consisted of four parts. In the first part, for each stimulus of *naively unrecognizable* type the following sequence of images was presented: naively unrecognizable bitonal Mooney figure - grey photo of the original, non-degraded image - grey photo of the same image with superimposed red contour lines - naively unrecognizable Mooney figure with superimposed red contour lines - naively unrecognizable Mooney figure (see Fig. 1). The duration of each image display in the sequence was 5 ​s. The second part was similar to the first one, but the duration of image display was reduced to 3 ​s. During the third and the fourth parts a participant was presented with the naively unrecognizable Mooney figures for 3 ​s and 800 ​ms respectively. The latter duration was identical to that in the main blocks of the experiment. The order of the presentations related to each *naively unrecognizable* stimulus was randomized. We further refer to the stimuli, that have been correctly identified as an object after this training procedure, as belonging to the group of *induced recognition* images.

For the MEG data analysis, we used data from participants for whom the following conditions were met: (1) both exemplars of *naively unrecognizable* faces/tools were actually unrecognized before training; (2) both exemplars within face/tool group became recognizable after training. The number of such participants was different for faces - 19 participants, and for tools - 23 participants. The intersection of these groups yielded 16 participants.

### 2.4 Auxiliary procedure to determine reaction time

To avoid movement and response artifacts in the MEG experiment, participants were asked to suspend their vocalized responses until after stimulus offset. In order to identify possible differences in recognition speed of *simply recognizable* and *induced recognition* stimuli and to account for their influence in latencies of evoked responses, we performed an auxiliary experiment. The reaction times (RT) were determined for 26 healthy volunteers, none of whom participated in the main experiment.

This procedure largely reproduces the procedure of the main experiment. The same set of stimuli were shown to the participants on a computer screen providing 8 ​× ​8° visual angle. We significantly reduced the number of images shown before the training procedure since, in this case, we only needed to determine which of the *naively unrecognizable* stimuli were actually unrecognized. The part following the training procedure comprised two 16-min blocks, similar to that of the main experiment.

The key difference – with respect to MEG data recording procedure – was that we asked participants to press a button as soon as they had recognized the object on the image. After responding, they were required to name aloud what they had seen in the picture. In order to discourage subjects from pressing a button before they recognized the object, we used backward mask that appeared in place of the image at the moment the button was pressed.

The RT results obtained in the auxiliary experiment are presented in Table 1.

**Table 1:** Reaction time of responses to *simply recognizable* Mooney figures and to those from the *induced recognition* set. The results were obtained from 26 healthy volunteers, none of whom participated in the main experiment.

Image class	Response time (Standard deviation), ms
*simply recognizable* faces	502(69)
*simply recognizable* tools	515(62)
*induced recognition* faces	578(79)
*induced recognition* tools	571(52)

### 2.5 MEG acquisition

Neuromagnetic responses were recorded with the helmet-shaped 306-channel sensor array (“Vectorview,” Neuromag Elekta Oy, Helsinki, Finland). In this study, data from 204 planar gradiometers were used for analyses. Prior to the MEG recording, the positions of HPI coils were digitized together with fiducial points using the 3D digitizer “FASTRAK” (Polhemus, Colchester, VT, United States) and were used to assess a subject’s head position inside the MEG helmet every 4 ​ms. Later, offline position correction procedure was applied to the recorded data to compensate for a head movement.

The spatiotemporal signal space separation method (tSSS) implemented by “MaxFilter” (Elekta Neuromag Oy software) was used to suppress interference signals generated outside the brain. An electrooculogram (EOG) was recorded using four electrodes placed at the outer canthi of the eyes as well as above and below the left eye. The MEG signals were recorded with a band-pass filter of 0.1–330 ​Hz, digitized at 1000 ​Hz, and stored for offline analysis.

### 2.6 MEG data preprocessing

The MEG data preprocessing used a combination of tools from SPM8 (Litvak et al., 2011), Fieldtrip (Oostenveld et al., 2011), as well as bespoke routines written in the MATLAB environment. The data were epoched from −500 ​ms prior to stimulus onset, until 1000 ​ms post-stimulus. Information from the experimenters’ recorded observations of the subjects’ responses was used to select trials with correctly categorized images. To suppress large initial repetition effects, we excluded the four initial presentations of each stimulus from 1st and 3rd blocks and the first presentations of each stimulus from 2nd and 4th blocks. Following this selection of epochs, the timeseries data were low-pass filtered at 24 ​Hz and baseline corrected using a −300 to −1 ​ms interval before stimulus onset. At the next step the data from the first and the second blocks, as well as those from the third and the fourth blocks were concatenated. The last step was done to provide two sessions of 75 presentations (or less following artifact trial rejection) for each stimulus. Hereinafter, these two sessions are used to define two independent conditions of presentation of every stimulus.

Following this, an independent component analysis (ICA) based artifact removal procedure was applied to the data separately for each subject. A set of eight random trials of each stimulus was drawn from both sessions to produce a set of 80 trials and this set was decomposed into independent components. We inspected time courses, spectra and topologies of the first 20 components and removed components comprising EOG, cardiac or muscle artifacts. After this, trials with residual artifact activity were rejected automatically using an algorithm that detects large deviations in response amplitude of the based on adaptive thresholds for each person and channel.

### 2.7 Source-localization and whole cortex atlas-based regions definition

All results presented in this study are based on source-space analysis. To reconstruct the evoked responses in source-space, we applied an anatomically constrained inverse problem solver, forcing the sources to lie on a tessellated mesh of the cortical mantle. The sources are considered as dipoles with fixed orientations, normal to the local curvature of the mesh. The meshes for every participant were obtained on the basis of individual high-resolution structural T1-weighted MRIs acquired on a 1.5 ​T Toshiba ExcelArt Vantage scanner (TR ​= ​12 ​ms, TE ​= ​5 ​ms, flip angle ​= ​20°, slice thickness ​= ​1.0 ​mm, voxel size ​= ​1.0 ​× ​1.0 ​× ​1.0 mm3). These structural scans were segmented, and the grey-matter segment was used to construct a continuous triangular mesh representing the neocortex using FreeSurfer software (Dale et al., 1999). The fiducial points digitized during MEG acquisition were then used to co-register the MEG and MRI spaces and individual meshes for every subject were downsampled to have 5002 vertices. These procedures – in addition to generation of an overlapping spheres forward model and sLORETA inverse operator calculation – were performed using the Brainstorm software (Tadel et al., 2011).

To register sources across subjects and to reduce a number of effective sources for the analysis – we used an automated labeling system for subdividing the cortex into gyri- and sulci-based regions, as implemented in the FreeSurfer toolbox. We use the Destrieux Atlas with 148 regions – covering almost the entire surface of the hemispheres (with an exception of medial wall structures which are lack of cortical sources). Some small regions from this atlas were combined into larger ones, while some elongated regions of temporal lobe were divided into posterior and anterior parts. This was done to ensure that every region in our analysis had comparable number of vertices that is necessary for comparing decoding accuracies between regions (Kozunov et al., 2018). The algorithm was very simple: we combine on the basis of anatomy a number of adjacent regions in such a way that the resulting region contained at least 50 vertices. The list of combined regions was the same for all participants. To divide, for each participant individually we found a mean coordinate for the region’s vertices along the anterior-posterior Y-axis, and the vertices with Y-coordinates exceeding this value were assigned to the anterior part, and those whose coordinates fell behind this value – to the posterior part of the respective region. Overall, the procedure resulted in 82 atlas-based regions. Although number of vertices assigned to the specific region might vary across subjects, each region represents the same anatomical structure.

A list of these regions – and the MNI coordinates of the seed vertices – are provided in Table 2. This table also contains the acronyms used to refer to the regions in the text.

**Table 2:** Complete list of the cortical regions examined in the present study with the acronyms and MNI coordinates of the seed vertices. To denote a region in right hemisphere only, the prefixes “r” are used in the text.

Region full name	Alias	MNI	Region full name	Alias	MNI
Frontal Pole	FPole	25, 61, −5	Entorhinal cortex	entorh	18,-20,-22
Superior Frontal	SF	18, 30, 44	Fusiform Gyrus	FG	37,-58,-19
Middle Frontal	MF	39, 40, 27	Medial Occipito-Temporal	MedOT	26,-53,-11
Inferior Frontal	IF	50, 20, 11	Collateral Sulcus anterior	ColSa	41,-25,-26
Orbital Frontal	OF	23, 28,-16	Precuneus	precun	6,-66, 47
Ventral Premotor	VPM	40, 2, 34	Angular Gyrus	AngG	49,-59, 40
Dorsal Lateral Premotor	DLPM	35,-10, 57	Inferior Parietal Gyrus	IP	57,-35, 31
Insula	insula	40, 6, 1	Superior Parietal Gyrus	SP	21,-65, 59
Cingular Anterior	CingA	3, 31, 23	Intraparietal Sulcus	IPS	30,-60, 43
Cingular Posterior	CingP	2,-26, 46	Parieto-Occipital Sulcus	POS	14,-69, 24
Postcentral Gyrus	postcen	43,-32, 49	Lunate Sulcus	LunS	30,-90, 9
Central Sulcus	central	41,-16, 51	Cuneus	cuneus	3,-83, 21
Temporal Pole	TPole	32, 12,-38	Superior Occipital Sulcus	SOS	27,-84, 25
Superior Temporal Gyrus anterior	STGa	52, 8,-10	Superior Occipital Gyrus	SOG	16,-93,34
Superior Temporal Gyrus posterior	STGp	55,-29, 16	Occipital Sulcus anterior	OSa	44,-73, −1
Middle Temporal anterior	MTa	65,-14,-20	Inferior Occipital G and S	IO	36,-90,-10
Middle Temporal posterior	MTp	67,-50, −2	Middle Occipital Gyrus	MO	39,-84, 19
Superior Temporal Sulcus anterior	STSa	52,-24, −6	Lingual Gyrus	LingG	8,-67, 0
Superior Temporal Sulcus posterior	STSp	51,-61, 17	Calcarine Sulcus	calcarine	17,-70, 7
Inferior Temporal anterior	ITa	58,-15,-28	Occipital Pole	OPole	15,-101,-4
Inferior Temporal posterior	ITp	50,-56,-20			

### 2.8 Region-based multivariate pattern classification analysis (RB-MVPA)

To characterize condition specific effects, we use a Region-based multivariate pattern classification (RB-MVPA) analysis used in the previous work (Kozunov et al., 2018). This approach applies linear discriminant analysis (LDA) - a standard method used to find a linear combination of features that separates two or more classes of objects - to the source-space responses in each brain region (see Table 2). Three principal components of each region’s vertex activities were used to train classifiers. This number of principal components provides a good compromise between preserving location specificity and sufficient dimensionality of the data feature space to discriminate between experimental conditions.

We performed RB-MVPA on the interval from 0 ​ms to 500 ​ms after stimulus onset. Before classification, we downsampled time courses (with ratio 10:1) to reduce the number of data points for further analysis (obtaining 51 time bins in total), and providing a 10 ​ms time resolution. To improve the signal-to-noise ratio, the set of 75 trials (or fewer after artifact rejection) for each condition was reduced to 10 pseudo trials by averaging a random selection of trials within this condition. Each pseudo trial was an average of five to eight trials. Conditions with less than 50 trials after artifact rejection were excluded from the analysis.

### 2.9 Cross-validation to assess classifier performance

Classifier performance was assessed with the following analyses. When classification performance was analyzed by comparing decoding accuracies or subject to RSA analysis, generalization was evaluated using cross validation. For each pairwise comparison, we select 18 pseudo trials used to train (nine from each stimulus class) and two used to test the classifier (one from each class). This procedure was repeated 100 times, over all possible combinations. Classifier performance was quantified in terms of accuracy; i.e., the proportion of correctly classified pseudo trials.

### 2.10 Temporal cross-category generalization analysis

The decoding approach can be extended to ask whether the neural code that supports decoding is stable or is dynamically evolving (King and Dehaene, 2014). Instead of applying a different classifier at each time point, the classifier trained at time T1 can be tested on its ability to generalize to time T2. This notion can be also extended for the cross-category analysis. If a classifier is trained in one condition and tested on its ability to generalize to another, the resulting generalization matrix may indicate that some of processing stages may remain unaffected whereas others may be accelerated, slowed, deleted, or inserted.

For obtaining of temporal cross-category matrices, we quantified classifier performance based on an accuracy of single separation of classes. Generalization of classifier performance, in this case, was achieved using a cross-decoding strategy which means the estimation of classifier performance rests on testing the classifier on samples that were not used to train it. This requirement is satisfied automatically when we train at one time and test at another or when we evaluate the ability of a classifier – trained on two groups of images at the same moment in time – to decode the two groups that are not within the training pair (e.g. train to decode *simply recognizable* faces vs *nonsense* and test on *induced recognition* faces vs *naively unrecognizable* faces). If at least one of the groups in the training pair coincides with the group in the tested pair (e.g. train to decode *simply recognizable* faces vs *nonsense* and test on *induced recognition* faces vs *nonsense)*, we excluded all pairwise comparisons in which both the training and testing pairs used the same image. According to these criteria, we classified all permitted combinations of images for a given training and testing pairs of groups and averaged results.

### 2.11 Representational similarity analysis (RSA) and representational dissimilarity matrix (RDM) profile

**Fig. 2:**
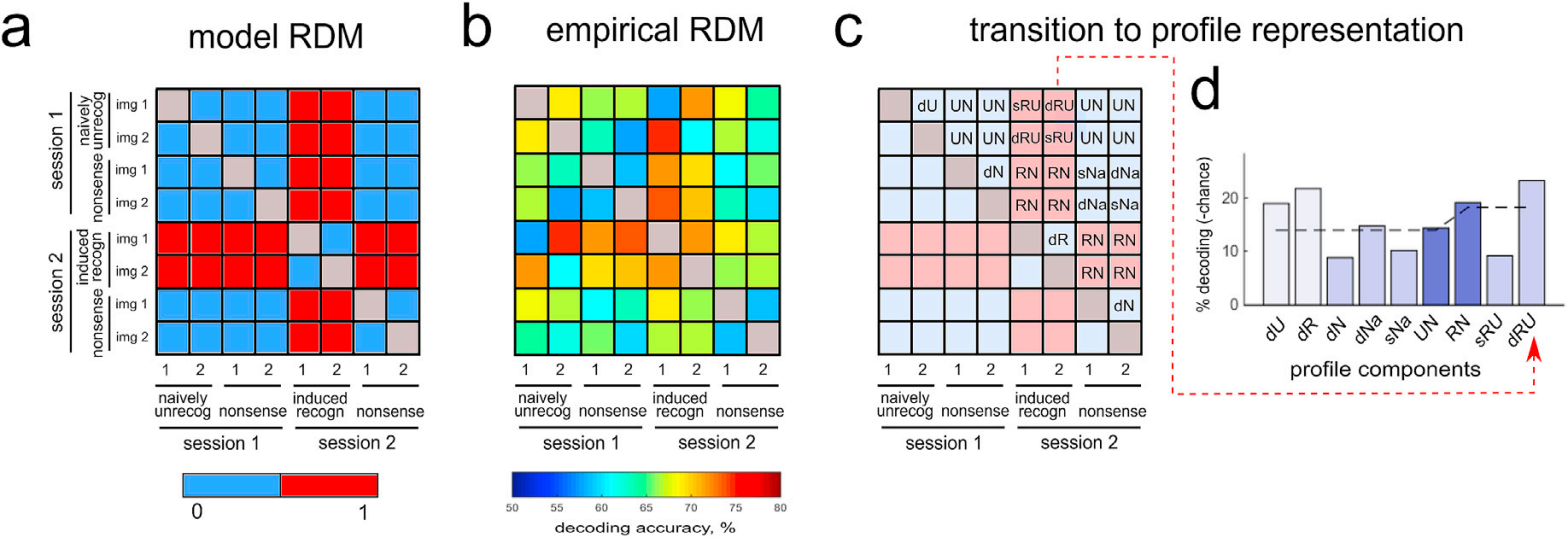
a - A model RDM used to extract recognition induced changes. This model required a higher dissimilarity between patterns of neural activity for stimuli across the recognized-unrecognized groups boundary than for within groups, wherein *naively unrecognizable* images (i.e. unidentified before training) were combined with *nonsense* images into one group. b - An example of an empirical RDM (averaged across subjects). For this example, we found a significant correlation with the model RDM, however in this representation, it is not easy to see any clear model structure, nor to ascertain which specific pairwise comparisons of the RDM give rise to the observed effect. c - Illustration of RDM transformation to so-called ‘profile’ representation: locations of RDM entries are combined into nine groups bound by a similar pairwise comparisons (please see Table 3 for the groups’ description). d - Example of the group averaged RDM profile (corresponding to the RDM on Fig. 2b). Red dotted line shows a correspondence between locations within the RDM and with those in the profile for one entry of the dRU (different exemplars of *induced recognition* vs *naively unrecognizable* faces/tools) component. The intensity of the bars’ color encodes the weight of the component based on the number of corresponding entries in the RDM. The first six profile columns correspond to low values of dissimilarity (0) in the model RDM and the last three ones correspond to high values (1). Dashed line indicates the weighted average across the first six and the last three component values.

To create spatiotemporal maps of neuronal responses induced by recognition of objects, we used RSA (Kriegeskorte et al., 2008). For this, we computed the Spearman’s correlation coefficients between the model (i.e., hypothesized) RDM and empirical RDMs for each region, each time point and each subject. The model RDM entailed a higher dissimilarity between patterns for stimuli across recognized-unrecognized group boundary than within groups, wherein *naively unrecognizable* images (before training) were combined with *nonsense* images in one group. These RDM were not intended to be definitive models of neural processing but represent condition specific effects by two values: 0 – low dissimilarity; or 1 – high dissimilarity (Fig. 2a).

RSA provides a powerful way to estimate empirical RDMs (Fig. 2b) by means of scalar values, given by the coefficients of correlation with the model RDM. Such a representation is degenerate because it does not consider which pairwise comparisons are important for correlation with the model. Here, we used an intermediate between complete RDM and scalar coefficient representation, which we refer to as an *RDM profile*. The RDM profile represents the pairwise classification results grouped (averaged) according to their role in the model (Fig. 2c). Table 3 presents these groups of comparisons (profile components) in the order of their location in the profile and indicates acronyms used to refer to them. Table 3 also lists contrasts between the condition specific components that we report in the Results section.

**Table 3:** RDM profile components.

Component number	Acronym	Number of entries in RDM	Group of pairwise comparisons	Utility of Contrast
1	dU	1	different exemplars of *naively unrecognizable* faces/tools	dR-dU is a recognition-induced increase in differentiation between exemplars within the same category
2	dR	1	different exemplars of *induced recognition* faces/tools	
3	dN	2	different exemplars of *nonsense* images within sessions	dNa-dN is a collateral index of changes between sessions not related to recognition
4	dNa	2	different exemplars of *nonsense* images across sessions	
5	sNa	2	same *nonsense* images across sessions	sNa is a direct index of changes between sessions not related to recognition
6	UN	8	*naively unrecognizable* faces/tools vs *nonsense* images	RN-UN is a gain in the decoding of faces/tools vs *nonsense* stimuli when they changed from unrecognizable to recognizable state
7	RN	8	*induced recognition* faces/tools vs *nonsense* images	
8	sRU	2	same face/tool image in *induced recognition* vs *naively unrecognizable* states	sRU-sNa is a decoding of the same face/tool, before vs after it became recognizable, while controlling for changes between session not related to recognition
9	dRU	2	different face/tool images in *induced recognition* vs *naively unrecognizable* states	

The first six profile components correspond to low values of dissimilarity in the model RDM and the last three ones correspond to high values. In the profile, we encode the weight of the component – based on the number of averaged pairwise comparisons – by the intensity of color. Dashed line indicates the weighted average across the first six and the last three component values (Fig. 2d).

### 2.12 Statistical testing

For statistical testing of the RSA results – as summarized by Spearman’s correlation coefficients – we applied a Fisher transformation. This ensures that their null distributions are close to normal and licenses the use of standard parametric statistical testing.

In the first part of the study, we present the results of spatiotemporal searchlight approach based on statistical parametric maps (SPM) of relations with the model RDM. Hypotheses – expressed in terms of the differences of the correlation coefficient from zero – were assessed at each spatiotemporal point with a univariate *t*-test. To adjust for multiple comparisons (82 regions ​× ​51 time bins) we used an FDR corrected threshold (q ​< ​0.05). In the second part of the study, statistical testing in the spatiotemporal regions of interest (ROIs) was performed using a full factorial analysis of variance (ANOVA) in a repeated measures design or a two-sample paired *t*-test.

To identify periods of significant (above-chance) classification in the temporal cross-category generalization procedure, we used non-parametric permutation tests and cluster-based correction for multiple comparisons (Maris and Oostenveld, 2007). To obtain a permutation distribution of maximal cluster size, we randomly shuffled the sign of subject-specific data points (10,000 times) and determined 2D clusters at the cluster definition threshold. Storing the maximal cluster statistic for each permutation sample yielded a distribution of the maximal cluster size under the null hypothesis. We report clusters as significant if they were greater than the 95% threshold constructed from the maximal cluster size distribution (i.e. cluster size threshold at p ​= ​0.05).

## 3 Results

### 3.1 Overview of recognition induced changes

**Fig. 3:**
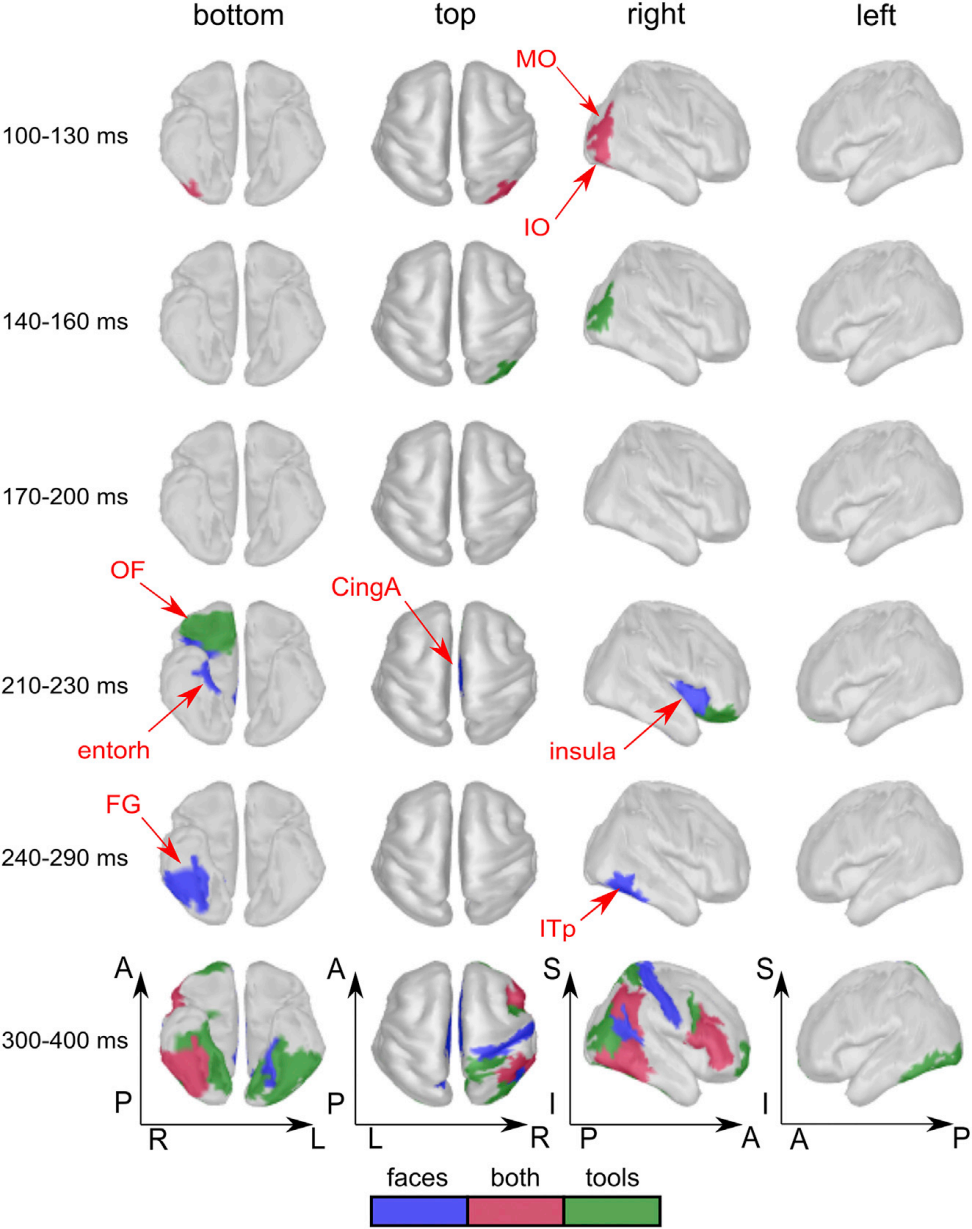
Grand average spatio-temporal map of changes in brain processing induced by a transition invoked during recognition of *naively unrecognizable* images. Successive time windows with persistent spatial distribution within each one are shown. Regions are only shown in the case for which at least half of time points within the specified windows have correlation coefficients that are significantly different from zero (False discovery rate corrected q-value ​< ​0.05). We color-code regions for which recognition induced changes were detected for specifically faces (blue), specifically tools (green) or for both categories (magenta). Red arrows indicate abbreviated names of regions with significant correlation.

**Fig. 4:**
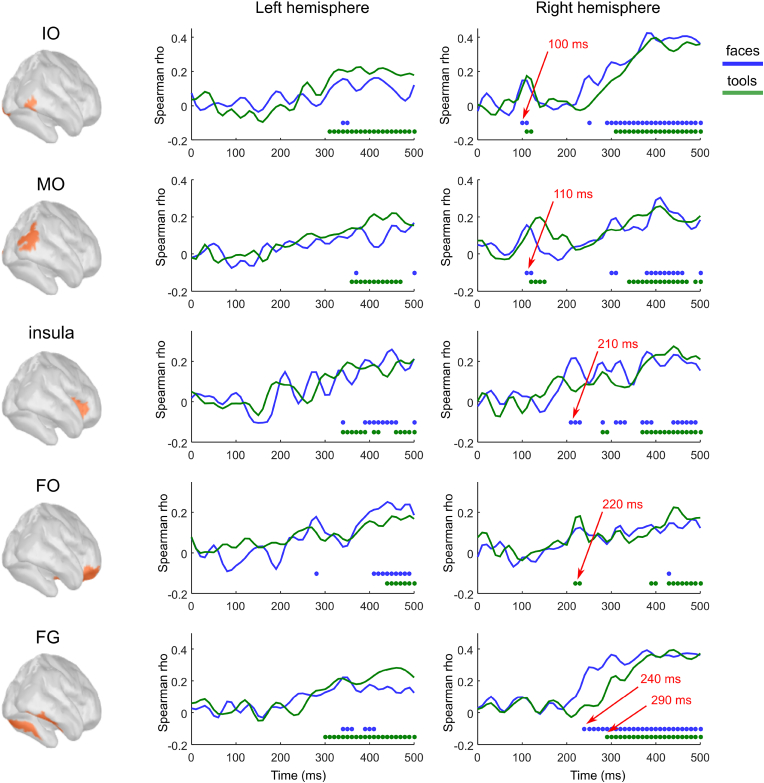
Grand average time courses of the correlation coefficients between empirical and model RMDs, which represents changes induced by the recognition of meaningful objects in the specified regions of the left and right hemispheres. Each row represents results for the region shown in the left-most panel. The color-coded markers under the curves indicate time points with significant correlations (FDR corrected q-value< 0.05). Both markers and time courses are color coded as follows: blue corresponds to faces; green to tools. Red arrows indicate characteristic time points corresponding to the onset of significant correlations.

Application of the RB-MVPA procedure – followed by RSA – created a spatiotemporal map of responses induced by the recognition of *naively unrecognizable* images (Fig. 3). The time courses of the correlation coefficients between the model and empirical RDM are presented in Fig. 4. The earliest effect (i.e., significant correlation between the model and empirical RDMs) was detected in extrastriate regions of the right hemisphere for both categories. For faces: in the *rIO* at the time window 100–110 ​ms and in the *rMO* at 110–120 ​ms (Fig. 4). For tools: in the *rIO* at the time window 110–120 ​ms and in *rMO* at 120–150 ​ms (Fig. 4). To test the location and category dependencies of this effect, we performed an ANOVA on the data that was averaged across overlapping time window 110–120 ​ms using the factors HEMISPHERE: right vs left, CATEGORY: face vs tool, and REGION: *IO* vs *MO*. The main effect of HEMISPHERE [F(1,15) ​= ​23.7, p ​= ​0.0002] confirmed the presence of a right hemisphere bias associated with the early recognition induced changes.

The second phase of recognition-induced responses started at 210 ​ms after stimulus onset and showed distinct spatial localizations in the brain for the two categories. For faces, significant correlations with the model RDM were found in the right *insula* at the time window 210–230 ​ms (Fig. 3, Fig. 4). Correlations were also found simultaneously in the right *CingA* and right *entorh* regions (Fig. 3) but lasted for shorter periods of time and with weaker correlation coefficients. For tools, recognition-induced changes were discovered in right *OF* area in the window 220–230 ​ms. Despite the difference in regions found for faces and tools, it is worth noting that they are all part of the brain’s value system (Chaudhry et al., 2009; Lane et al., 1997; Morris et al., 1998; Winston et al., 2002; Anderson et al., 2003; Phan et al., 2004).

Within the later time window 240–290 ​ms, we found recognition induced changes in the right *FG* and the adjacent right *ITp* for face but not for tool images (Fig. 3, Fig. 4). To confirm that this effect in the right *FG* was face specific, we performed an ANOVA on the values averaged across this time window with factors HEMISPHERE: right vs left, and CATEGORY: face vs tool. The main effect of CATEGORY [F(1,15) ​= ​9,52, p ​= ​0.0075] was found significant, as well as interaction HEMISPHERE x CATEGORY [F(1,15) ​= ​5,03, p ​= ​0.04]. This interaction indicates that this face specific recognition effect arose in the right hemisphere.

After 300 ​ms, widely distributed regions of parietal, temporal, and prefrontal cortices evinced recognition effects for both object categories (Fig. 3). Some of these regions are not shown in Fig. 3 because of long interval of averaging chosen for the late period, which is not of the primary interest of the study. A similar network of regions was previously observed for *simply recognizable* classes of images (Kozunov et al., 2018), where the effect emerged earlier at about 250 ​ms after the stimulus onset. The difference in latency of 50 ​ms can be related to the differences in the reaction times of behavioral responses to *simply recognizable* and *induced recognition* types of images (see Table 1).

### 3.2 Profile analysis shows distinct processing characteristics of successive phases

RSA conveniently summarizes relations to the model RDM by means of a single number (a correlation coefficient), which acts to obscure the details of pairwise comparisons (separate entries in the RDM). In order to analyze the results of the RSA in more detail – and to show the processing characteristics of different phases of recognition induced changes – we used an intermediate between complete RDM and a scalar representation which we designated as an RDM profile (Fig. 2d).

**Fig. 5:**
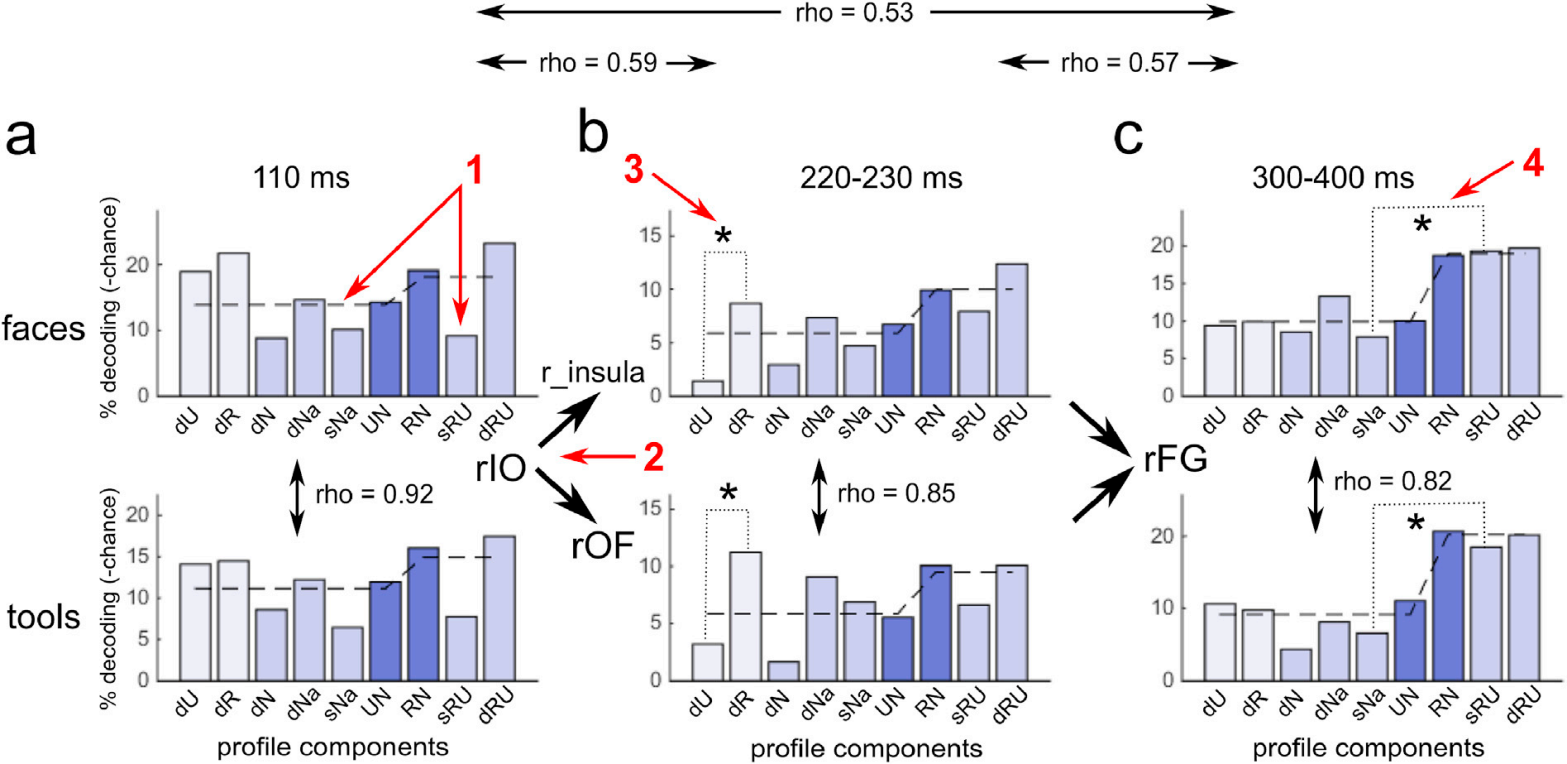
Grand average RDM profiles averaged across the specified time windows in the regions for which RSA detected recognition induced neural changes. Profiles for faces and tools are shown in the top and bottom rows respectively. a - The early phase of recognition induced changes, b - intermediate phase, c - late phase. The correlations of face vs tool profiles within all phases are significant. The correlations across phases (averaged across categories) are nonsignificant. Numbered red arrows point to the profile’s characteristics of interest: 1 - during the early phase decoding accuracy is equally poor for the same *nonsense* images across sessions (sNa) and for the same images undergoing *naively unrecognizable* - *induced recognition* transition (sRU); 2 - during the intermediate phase the profiles in the two different regions of value system - right *insula* for faces and *rOF* for tools - are correlated; 3 - during the intermediate phase, recognition increases differentiation between exemplars belonging to the same category; 4 - during the late phase, decoding accuracy is significantly greater for the same images in recognizable vs unrecognizable conditions (sRU) than for the same *nonsense* images across sessions (sNa).

Fig. 5 displays the group averaged RDM profiles for both face and tool images corresponding to the 3 successive phases of recognition effects. The presented regions and time windows were selected on the basis of the RSA. For the earliest phase, among two regions we chose to present *rIO* (similar results were found when analyzing the *rMO* region). For the intermediate phase, the right *insula* was selected for faces as it had the greatest correlation coefficients and the highest significance values among the three regions identified by RSA. The only significant region – right *OF* – was selected for tools. For the last phase, we chose the *rFG* region as it was the region with the most persistent effect after 300 ​ms and it demonstrated the greatest average values of correlation for both faces and tools. Time windows of overlapping intervals exhibiting significant correlations for faces and tools for each phase were chosen (for the late phase we arbitrarily selected the upper limit of the interval at 400 ​ms).

First, we computed Spearman correlation between profiles for faces and tools within the same phase and across successive phases. The correlations within every phase were significant: earliest - rho ​= ​0.92, p ​= ​0.0015; intermediate rho ​= ​0.85, p ​= ​0.007; late - rho ​= ​0.82, p ​< ​0.01. Whilst, correlations across phases (averaged by categories) were not significant: earliest-intermediate rho ​= ​0.59, p ​= ​0.1; earliest-late rho ​= ​0.53, p ​= ​0.15; intermediate-late rho ​= ​0.57, p ​= ​0.12. Since the absence of significant correlations does not guarantee the significance of differences between profiles, we performed ANOVA with factors PROFILE: 1–9 components, CATEGORY: face vs tool, and PHASE: early, intermediate, and late. A significant interaction PROFILE x PHASE [F(16,240) ​= ​4.51, p ​< ​0.0001] and a lack of interaction PROFILE x CATEGORY [F(8,240) ​= ​0.92, p ​> ​0.5] provides complementary results to suggest that (1) the phases are not only separated in time but also differ in processing characteristics; (2) the profiles for both categories were similar within each phase. It is worth noting that for the intermediate phase, the profiles in right *insula* for faces and in right *OF* for tools were correlated. There was no significant correlation within this phase for either *insula-insula* nor *OF-OF* pairs, which speaks in favor of the hypothesis that *rOF* region plays a similar role in tool processing as the right *insula* for faces.

Having established the uniqueness of each phase of object recognition, we next explored their properties. The early phase is characterized by a recognition-induced decoding gain for the faces/tools versus *nonsense* images (RN ​> ​UN on Fig. 5a; for faces t(18) ​= ​3.08, p ​< ​0.01; for tools t(22) ​= ​3.1, p ​< ​0.01). This effect was evident without significant decoding of the same images before and after they become recognizable, controlling for differences that were not related to recognition (sRU ​= ​sNa on Fig. 5a; for faces t(18) ​= ​0.88, p ​> ​0.35; for tools t(22) ​= ​0.54, p ​> ​0.54; and they are the lowest among all columns). This means that moderate categorical structure emerged in this region, while maintaining a unique spatial pattern for each stimulus.

The distinctive feature of the second phase is that distinguishability between stimulus categories emerged at the same time as there was a significant increase of within-category identification for recognizable images (dR ​> ​dU on Fig. 5b). We show that for processing of faces in the right *insula*, there is a significant difference in the decoding of different examples within the group before (dU) and after they become recognizable (dR): t(18) ​= ​2.17, p ​< ​0.05. And similarly for tools in the right *FO* region: t(22) ​= ​2.34, p ​= ​0.03. The coincidence of this result for faces in the *insula* as well as for tools in the *OF* area reinforces the conclusion – drawn from the previous correlation analysis – that these distinct brain regions of the value system are likely to play a similar role for the two different categories of stimuli.

The last phase of recognition induced changes – starting after 300 ​ms – are characterized by an ideal category structure, when decoding of all stimulus pairs across recognized-unrecognized boundary was higher than within group (the first six profile components corresponding to low values of dissimilarity in the model RDM have lower accuracy of decoding than the last three ones corresponding to high values). In particular, during this phase – for both categories – decoding of the same images in recognizable *vs* unrecognizable conditions in the right *FG* region (sRU on Fig. 5c column) became significantly higher than decoding of the same nonsense images in the 1st vs 2nd sessions (sNa) serving as a control for recognition-independent changes between sessions. For faces: t(18) ​= ​4.4, p ​< ​0.001; and for tools: t(22) ​= ​6.55, p ​< ​1e-5.

### 3.3 The early effect is power-dependent, but the intermediate effect is pattern-dependent

The distinctive properties of the early and intermediate phase’s RDM profiles speaks to the hypothesis that the early effect was caused by an augmentation of the same processing that was present for unrecognized stimuli, while the effect during the intermediate phase was the result of experience-dependent changes in the structure of processing. To confirm this, we carried out the following analysis in the *rIO* and right *insula* regions for face stimuli.

**Fig. 6:**
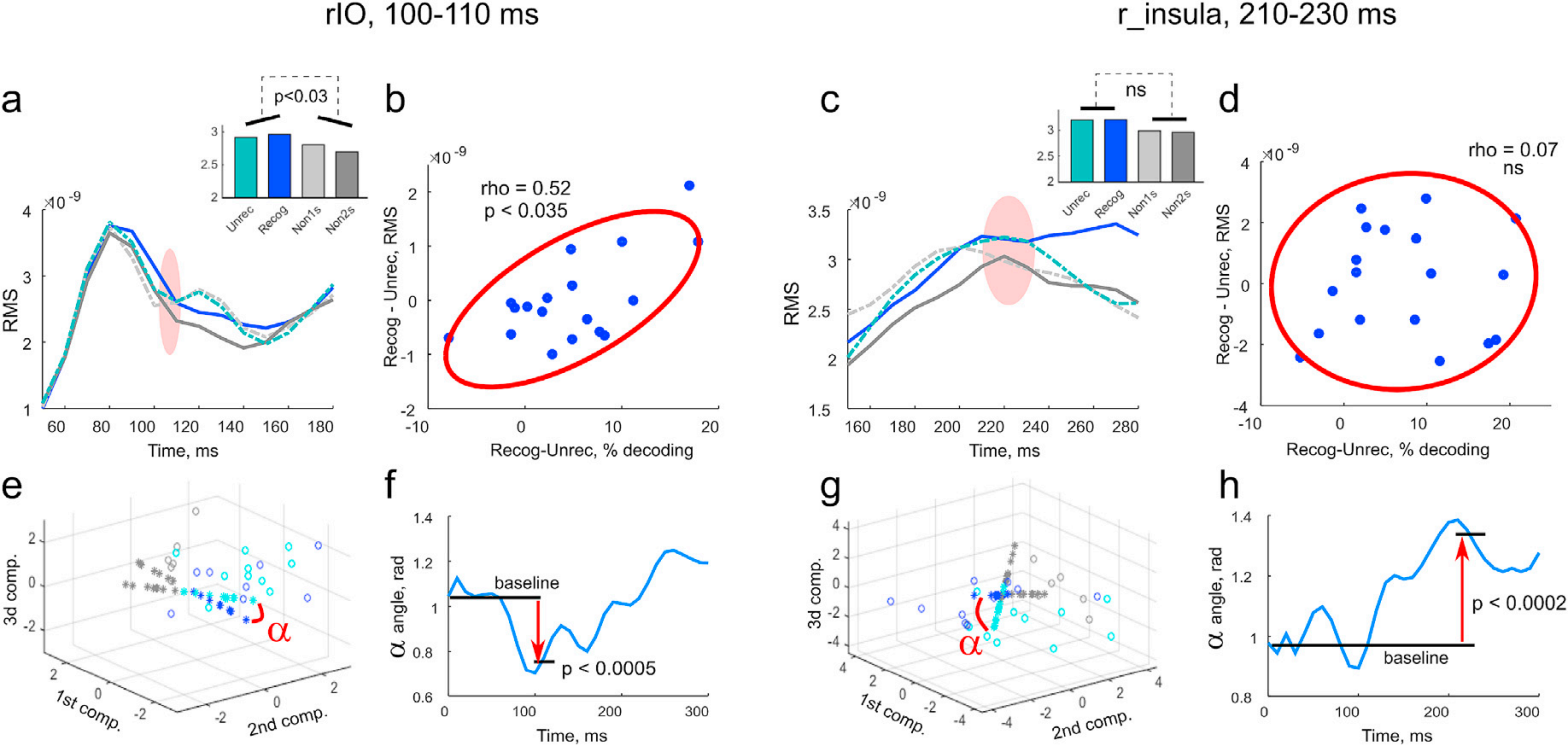
The early effect is power-dependent, the intermediate effect is pattern-dependent. a - Grand average time courses of the root-mean squared (RMS) power in *rIO* region for *induced recognition* faces (solid blue), *naively unrecognized* faces (dashed cyan line), *nonsense* images of the 2nd session (dark grey), *nonsense* images ofthe 1st session (dashed grey line). The same color coding is used for the insert bar plot. The bar plot represents mean values of the RMS power within the time window 100–110 ​ms (the early effect) which is designated on the main plot by the pink shadow ellipse. b - Scatterplot showing a significant correlation between the increase in decoding versus the increase in the RMS power when averaged across the time window 100–110 ​ms in *rIO* region. The increase in decoding was determined as accuracy of *induced recognition* vs *nonsense* decoding minus accuracy of *naïvely unrecognizable* vs *nonsense* decoding. The increase in the RMS power was determined as *induced recognition* power minus *naïvely unrecognizable* power. The red ellipse indicates bounds at 1.5 std; c and d - same as (a and b) but for right *insula* during the intermediate effect (210–230 ​ms). Neither the changes in the power or decoding-power correlation were found to be significant. e − 3D representation of joint feature space for MVPA. Empty circles represent samples used in the classification: blue - *induced recognition* faces, cyan - *naively unrecognizable* faces, grey - *nonsense* images. Asterisks (with the same color coding) represent these samples projected onto the normals of the planes found by LDA that respectively separate the comparisons between *induced recognition* vs *nonsense* and *naively unrecognizable* vs *nonsense* images. The angle alpha between these normals measures how different the patterns are - the smaller alpha the more similar the patterns. An example measurement of the alpha in the *rIO* within the 100–110 ​ms interval for one choice of stimuli and one participant is shown. f - Grand average timecourse of the alpha angle in the *rIO* region. The small value of alpha in the time window 100–110 ​ms indicates the high similarity of activity patterns before and after the stimulus became recognizable. A baseline level was calculated from activity in the −200 to 0 ​ms interval and is shown here with a time shift for visualization purpose. g - The same as for (e) but for the activity in the right *insula* within the 210–230 ​ms interval for one example of stimuli within a participant. Note a bigger angle alpha (than seen in e) which corresponds to a larger displacement of *induced recognition* patterns in the MVPA feature space in relation to *naively unrecognizable* ones. h - Grand average time course of alpha in the right *insula*. The high value of alpha for the effect within the intermediate phase time window indicates that recognition induced a significant change of patterns of activity at this phase when compared to chance level.

Initially, we analyzed the root-mean squared (RMS) power of the three principal components for a given region that were used to train LDA classifiers. For the *rOI* area and within the 100–110 ​ms window (taken as the interval of significant correlation with the model RDM and averaged across the window) we computed an ANOVA with the factors SESSION: first vs second, and GROUP: faces vs *nonsense*. This revealed a significant interaction between these two factors: [F(1,18) ​= ​6,2, p ​< ​0.025]. This indicated that the power of *induced recognition* faces compared to *naively unrecognizable* images was not subject to a suppression effect (i.e., they demonstrated a subthreshold enhancement: see the insert on Fig. 6a for the difference in means), which was expressed for *nonsense* stimuli between the sessions. Moreover, we have found a significant Spearman’s correlation between the increase in the decoding (*induced recognition* vs *nonsense* decoding minus *naively unrecognizable* vs *nonsense* decoding) and the increase in the power (RMS of *induced recognition* minus RMS of *naively unrecognizable*): rho ​= ​0.52, p ​< ​0.035 for the early phase effect (Fig. 6b). This suggested that the difference in decoding between the *naively unrecognizable* and *induced recognition* images was linearly related to an increase in power.

A similar analysis of the activity in the right *insula* at the time window of intermediate effect did not show an increase of the power for *induced recognition* faces compared to *naively unrecognizable* images processing, nor a significant correlation between the changes in the power and decoding of the stimuli when they become recognizable (Fig. 6c and d).

A complementary characterization of the RMS power of regional activity is afforded by the relative contributions (a pattern) of the three principal components, which may be represented by a direction (unit vector) in the 3D (according to the number of principal components) MVPA feature space. The angle between the two directions can be used as a measure of power independent pattern similarity. Here, we were interested in the (dis)similarity of two patterns (before and after the stimulus becomes recognizable) not in general but in the context of their differential ability to be decoded from a third (*nonsense*) stimuli. This leads us to use the angle (designated as alpha) between the normals to the two planes, found by the LDA for the best separation of the same stimulus in recognizable and unrecognizable conditions from a *nonsense* stimulus (Fig. 6e and g). While the directions of the normals are subject to considerable variability – depending on the selected stimuli and participants – the angle between them is independent of this variability and can be averaged across the group and subjected to statistical analyses.

Fig. 6f shows the group averaged time course of the angle alpha for the *rIO* region. When alpha is averaged across the time window of the early effect, we found a significantly lower value than the alpha averaged across the baseline period (from −200 to 0 ​ms): t(18) ​= ​4.24, p ​< ​0.0005. We assume the activity in the baseline can be considered as a chance value of similarity between patterns. Then this result indicates that during the early phase similarity between activity patterns before and after the stimuli become recognizable was significantly higher than that of chance level. In contrast to this, Fig. 6h shows the group averaged time course of the angle alpha for the right *insula* region. For the time window of intermediate phase effect we found a significantly higher value of alpha than during the baseline period: t(18) ​= ​4.81, p ​< ​0.0002. This result indicates that during the intermediate phase there was a significant change in activity patterns that occur independently of differences in power.

Altogether, these results indicated that early effect in the *rIO* region were largely due to an increase in power of neural activity and occurring without any substantial change in the activity pattern. We interpret this as a strengthening of the same processing that was present for unrecognized stimuli. In contrast, the intermediate phase effect in the right *insula* consisted of changes in pattern of neural activity with no significant change in power. This suggests that the effect in *insula* arose due to a transition to a new configuration in the value space that determines a new salience structure.

Following a similar analysis, we obtained the same pattern of results for tool images in extrastriate regions but did not find significant effects for tool images in r*FO* area. We suggest that this may be explained by the impoverished signal-to-noise ratio of MEG recoding from frontal regions for the intermediate phase time window.

### 3.4 Temporal cross-category generalization testified a time shift of “face-specific N170m” component for *induced recognition* faces

Results obtained with RSA suggest that a recognition induced effect in *rFG* for faces appeared only at 240 ​ms after stimulus onset, which is 30 ​ms after that found in *insula*. In section 3.3 it was noted that at the same time, an *insula* effect was power independent, making it implausible that it is associated with the well reported “face-specific N170” component. This suggests that perception of *induced recognition* faces occurred either without this component or that it is expressed at a later time. To test these possibilities, we performed temporal cross-category generalization analysis.

**Fig. 7:**
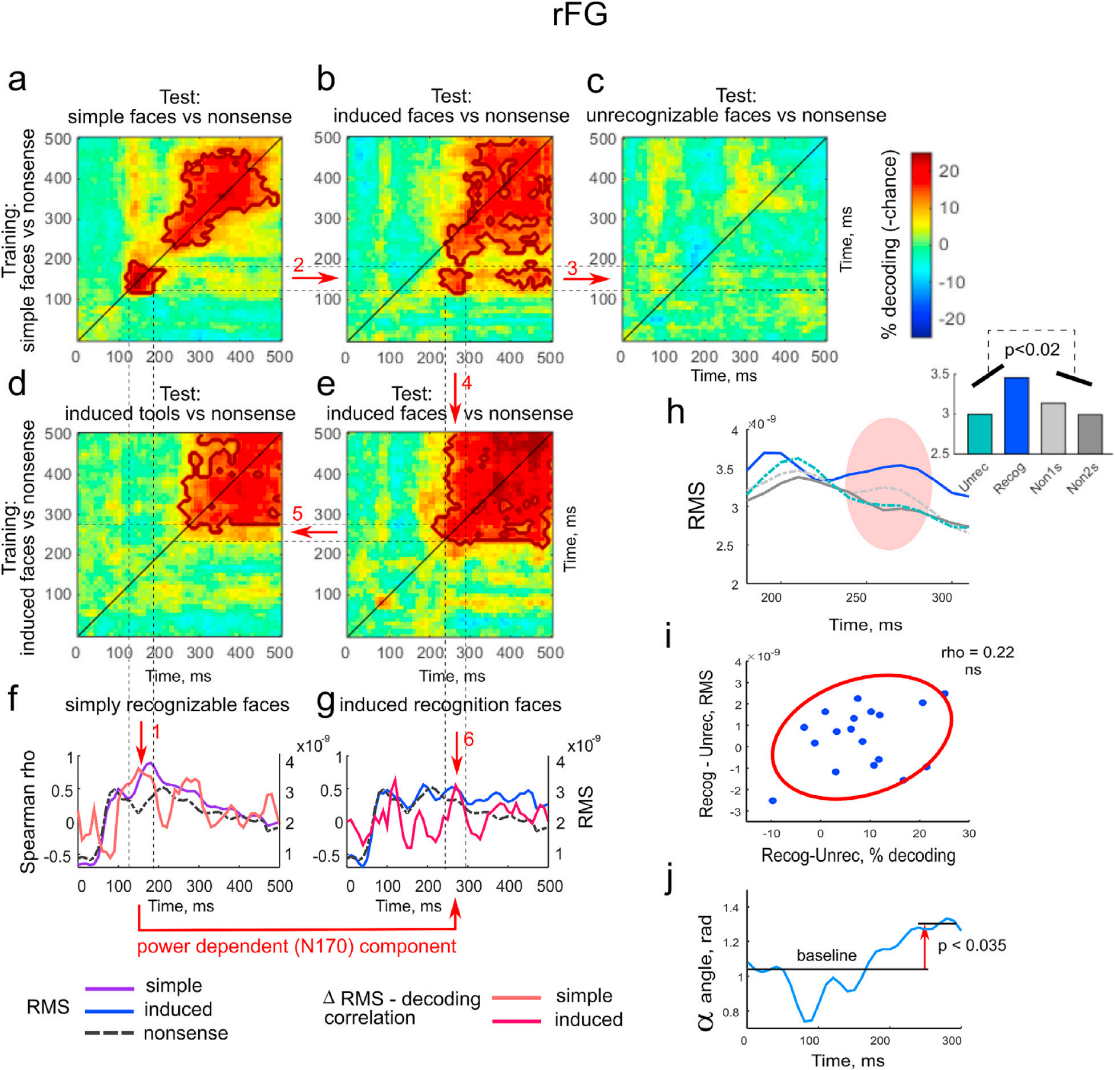
Temporal cross-category generalization analysis. We investigated the possibility that for different image classes, similar processing patterns are shifted in time. a-e − Grand average results of the temporal cross-category generalization in the right *FG* region are presented in heat maps. Please refer to the legend to interpret colors and to the axis labels for each individual plot to inquire what were used as the training and testing pairs (on Y and X axes respectively; the same training classes are presented along the rows). Solid brown contours designate clusters of significant decoding. The solid diagonal line from bottom left to top right corner on each plot indicates training and testing at the same time. Dotted lines are drawn to help match time intervals on different plots. f-g – Grand average time courses of RMS power with the correlation between the increase in power and decoding superimposed: f - for *simply recognizable* faces and *nonsense* stimuli; g - for *induced recognition* faces and *nonsense* stimuli. Red arrows with indices indicate: 1 - the cluster of significant cross-decoding for *simply recognizable* faces at 140–180 ​ms is power-dependent; 2 - classifiers trained in this interval for *simply recognizable* faces decode *induced recognition* faces from *nonsense* at 240–290 ​ms; 3 - but will not ever decode *naively unrecognizable* faces; 4 - within the interval 240–290 ​ms classifiers trained to decode *induced recognition* faces are generalized within this class; 5 - but are not generalized to decode *induced recognition* tools; 6 - the cluster at 240–290 ​ms for *induced recognition* faces is power-dependent. h-j - The same power vs pattern changes analysis as presented on Fig. 6 but for the *rFG* region: h – Grand average time courses of RMS power. The bar plot represents mean values for the time window 240–290 ​ms; i - Scatterplot of the increase in the decoding (*induced recognition* vs *nonsense* decoding minus *naively unrecognizable* vs *nonsense* decoding) against the increase in the power (RMS of *induced recognition* minus RMS of *naively unrecognizable*) averaged across the time window 240–290 ​ms in *rIO* region. Please note, this plot does not show a significant correlation between a recognition induced increase of decoding and an increase of power as opposed to the relation of decoding and power difference between *induced recognition* and *nonsense* of which the time course is presented on (g). j – Grand average time course of the pattern similarity measure. The alpha angle averaged over the time window 240–290 ​ms is significantly larger than that observed for the baseline period which speaks to recognition induced change of activity patterns.

We started the temporal cross-decoding generalization analysis within *simply recognizable* face group in the right *FG* region. When groups for training and testing are the same, this approach should provide similar results (on the main diagonal where training and testing occur at the same time) as RSA for the correlation with categorical structure model RDM (they both require differences between groups generalized for the exemplars within groups). In Fig. 7a two clusters of significant cross-decoding can be seen: first in the window 140–180 ​ms, second in the window 250–450 ​ms. This result accords with an RSA analysis reported in our previous work (Kozunov et al., 2018). The first cluster of decoding corresponds to the power-dependent “face-specific N170m” component because it conformed to the increase of power for *simply recognizable* faces as compared with *nonsense* stimuli: t(18) ​= ​2.93, p ​< ​0.01. Moreover, this difference in power was correlated with accuracy of decoding of *simply recognizable* faces vs *nonsense* stimuli: rho ​= ​0.71, p ​< ​0.001 (Fig. 7f).

Pursuing this, we found that classifiers trained to decode *simply recognizable* faces vs *nonsense* at the time of N170m component were able to decode *induced recognition* faces vs *nonsense* at the later time window 240–290 ​ms (Fig. 7b). The exact time limits of this interval were testified by their consistency with boundaries of face-specific effect in the *rFG* for the time-resolved RSA (Fig. 3, Fig. 4). Importantly, it was shown that these classifiers did not decode *naively unrecognizable* faces vs *nonsense* in either the specified 240–290 ​ms window nor in any of the remainder of the analyzed times (Fig. 7c). This finding predicts an emergence of a novel, recognition induced patterns of activity. Additionally, in the interval 240–290 ​ms classifiers trained to decode *induced recognition* faces vs *nonsense* were generalized within this group (Fig. 7e) but were not generalized to decode *induced recognition* tools from *nonsense* images (Fig. 7d), not even with a delay. After 290 ​ms such a generalization from *induced recognition* face to tool stimuli was present without a delay. This reinforces the view that the process extracted for *induced recognition* faces in the interval 240–290 ​ms in the right *FG* was face-specific.

However, this component was only partially dependent on power. On the one hand, the time course of the correlation of the accuracy of decoding (*induced recognition* vs *nonsense* decoding) and difference in power of processing of *induced recognition* minus *nonsense* images demonstrated a pronounced rise in this window (Fig. 7g) at the peak of which this correlation reached significance: rho ​= ​0.51, p ​< ​0.03. Moreover, an ANOVA computed across the window averaged values with the factors SESSION: first vs second, and GROUP: *induced recognition* faces vs *nonsense*, showed significant interaction between the factors: [F(1,18) ​= ​6,71, p ​< ​0.02] and demonstrated a relative increase of power for this recognition induced effect (insert on Fig. 7h).

In contrast, the correlation of the accuracy of decoding and difference in power for the time window averaged values (as opposed to peak value) did not reach significance: rho ​= ​0.41, p ​> ​0.09. Additionally, the correlation (analyzed in section 3.3 and that cannot be applied to *simply recognizable* stimuli) between the recognition-induced increase in the decoding (*induced recognition* vs *nonsense* minus *naively unrecognizable* vs *nonsense*) and the recognition induced increase in the power (*induced recognition* minus *naively unrecognizable*) was not significant (Fig. 7i): rho ​= ​0.22, p ​> ​0.37. Finally, we found that unlike the early effect in *rIO/rMO*, the effect in *rFG* also included those indicating pattern changes. For the time window 240–290 ​ms we found a significantly higher value of the alpha angle than that found for the baseline period (Fig. 7j): t(18) ​= ​2.81, p ​= ​0.035. Collectively, this suggests that this effect in the *rFG* was partly due to an increase in power and partly to a change in pattern of activity.

## 4 Discussion

### 4.1 Summary of findings

In this study, we characterized the changes in spatiotemporal patterns of neural activity underlying recognition of Mooney figures that depends upon previous experience. To this aim, we applied RB-MVPA followed by RSA to MEG data collected during a task that induced object recognition effects. Recognition induced changes were first seen at 100–120 ​ms, for both faces and tools. These early effects in the right inferior and middle occipital regions were characterized by an increase in power in the absence of any activity pattern reorganization. We interpret these findings as signatures of an enhancement of a pre-existing sensory saliency maps corresponding to conspicuous spatial features.

Following 210 ​ms post-stimulus onset, a quite different type of recognition effect emerged. In the category-dependent regions of brain’s value system, namely, *insula*, *entorhinal cortex* and *anterior cingulate* of the right hemisphere for faces and right *orbitofrontal cortex* for tools, patterns of neural activity demonstrated dissimilarity for images across recognized-unrecognized group boundary. This effect emerged with a concomitant differentiation between exemplars within the group of recognized images which suggests an increase in sensitivity to distinct visual features that are relevant for categorization (Folstein et al., 2013). Moreover, for faces, we found that the effect in the *insula* can be derived from changes in the pattern of neuronal activity without an overall power alteration in the region. Collectively, these findings suggest that responses in the intermediate phase of peristimulus time are likely to be attributed to an experience-dependent reorganization of value-based salience.

Finally, we found that during the perception of *induced recognition* face stimuli, a face-specific process in the right *fusiform gyrus* was delayed to 240–290 ​ms, relative to the well-known N170m component. This indicates that face selective processing may depend on the value system changing the salience of lower-level visual features. However, even an *induced recognition* face perception relies more on *fusiform gyrus* processing compared with *induced recognition* tools for which this effect at 240–290 ​ms was not detected.

### 4.2 The effect found in the initial phase is likely due to repetition enhancement of neuronal responses accompanied by gradual improvement of image recognition

Over the last few decades, a large number of studies have been conducted using disambiguation of Mooney figures. Various neuroimaging techniques have been used in these experiments: positron-emission tomography (PET) (Dolan et al., 1997), functional magnetic resonance imaging (fMRI) (Hegdé and Kersten, 2010; Ludmer et al., 2011; Gorlin et al., 2012; Van Loon et al., 2016; González-García et al., 2018), electroencephalography (EEG) (Jemel et al., 2003; Goffaux et al., 2004; Martens et al., 2012; Minami et al., 2014; Samaha et al., 2016), MEG (Urakawa et al., 2015; Flounders et al., 2019), repetitive transcranial magnetic stimulation (rTMS) (Giovannelli et al., 2010) and even single neuron recording in a monkeys (Tovee et al., 1996). In these studies, recognition effects were found in low-level visual areas (Gorlin et al., 2012; Van Loon et al., 2016), ventral occipito-temporal regions (Tovee et al., 1996; Dolan et al., 1997; Gorlin et al., 2012; Hegdé and Kersten, 2010; Martens et al., 2012; Urakawa et al., 2015; Van Loon et al., 2016), parietal cortex (Dolan et al., 1997; Giovannelli et al., 2010; Minami et al., 2014; González-García et al., 2018), and frontal cortex (Martens et al., 2012; González-García et al., 2018). Localization information has been drawn largely from fMRI based studies, whilst time-resolved EEG and MEG has provided temporal information about processing of disambiguated images. Recognition induced effects have been found at 120 ​ms (Urakawa et al., 2015; Samaha et al., 2016), at the peak of N170 component (Jemel et al., 2003), around 200 ​ms (Goffaux et al., 2004), after 250 ​ms (Minami et al., 2014), after 500 ​ms (Flounders et al., 2019), after 750 ​ms (Martens et al., 2012).

Such a wide range of areas and time intervals, with a number of inconsistencies among different studies, emphasizes the need to consider the details of each experimental paradigm. The testing time of the disambiguation effect varies; either, immediately after a period of training (Jemel et al., 2003; Minami et al., 2014; Chang et al., 2016), or later in time (e.g. after a week and/or after a wide set of intervening images was shown) (Goffaux et al., 2004; Ludmer et al., 2011; Martens et al., 2012). This factor determines the different contributions of priming, repetition effects, and familiarity. For example, the early disambiguation induced effect at 120 ​ms (Urakawa et al., 2015; Samaha et al., 2016) has been previously reported when a priming effect was prevalent and thus the effect was dominated by suppression of neuronal activity. In our study, we precluded priming effects and reduced the impact of repetitions in order to emphasize the long-term effect of familiarity, which was the primary focus of this experiment. To ensure this: (1) we performed training and testing in two different sessions separated by at least 5 ​min break; (2) during testing session, we arranged the presentation of both the primary and auxiliary stimuli in a pseudorandom order – so that on average there were 10 presentations of other images between two presentations of the same stimulus; and (3) we excluded the initial presentations of each stimulus in all blocks from the analysis (see Methods section 2.6).

Nevertheless, the results from our analysis of evoked responses (which requires averaging across multiple presentations of the same stimuli) are affected by repetition effects. One very reproducible finding in neuroimaging studies is that repeated stimuli elicit lower responses than novel stimuli. In an apparent contradiction, some studies have reported the opposite effect – increases in response to repeated stimuli and across many of the same brain regions. These latter enhancement effects are typically obtained when stimuli have been degraded (Turk-Browne et al., 2007; Kozunov et al., 2018) or are unfamiliar (Henson et al., 2000), such that repetition of a stimuli gradually induces their recognition by the observer. In support of this, it has been shown that repeating the same, initially novel and poorly recognizable images of scenes (for up to five repetitions) can enhance BOLD-responses (Müller et al., 2013). Additional repetitions resulted in a progressive attenuation of neural responses, in what is thought to indicate a more efficient representation of the now easily recognizable stimulus. Further, it has been shown that for non-trivial stimuli, which are processed at several levels (spatial and semantic), multiple intervals of repetition effects can occur (Stefanics et al., 2018; Henson et al., 2008; Recasens et al., 2015). It is notable that a common pattern of results has been obtained in all these studies; namely, that the repetition enhancement effects occur at later intervals than suppression effects.

In our study, the earliest effect at 100–120 ​ms in the extrastriate regions of the right hemisphere was due to a relative increase of *induced recognition* stimuli activity that occurred at the same time at which a suppression effect was seen for the presentation of *nonsense* images. A repetition suppression in the time window of the P1 component – and in the absence of top-down influences that might modulate repetition-related neural activity – has been repeatedly shown in several studies (Stefanics et al., 2018; Henson et al., 2008). This finding can be interpreted as a minimization of prediction error (i.e., a sharpening) at the level of visual feature extraction. In contrast, the repeated presentation of degraded stimuli in the experiment presented here acted to increase the activity in a time-window partially overlapping with the P1. This is most likely because the learning enhances the selectivity for relevant shape-dependent cues in lateral occipital complex (Gillebert et al., 2009; Lerner et al., 2008; Folstein et al., 2013). Moreover, a recent study (Brants et al., 2016) has shown that (extensive) category learning strengthens the pre-existing functional selectivity of the relevant distinctive shape-based features. Since we also found that – for the early effect – there was a strengthening of the same activity patterns that were present for unrecognized stimuli, we conclude that this effect is most likely not due to a sudden transition to meaningful object recognition but was instead caused by gradual enhancement of recognizability.

### 4.3 Experience-dependent perceptual prior originates from a familiarity of integrated spatial and value-space saliency map

The sudden disambiguation of a Mooney figure can be associated with the experience-dependent change of the salience of particular visual features (Ludmer et al., 2011). We consider here salience as an interface between spatial attention (both exogenous and endogenous) and emotional states (Compton, 2003). This interface reconciles influences of conspicuous visual features (Rutishauser et al., 2004; Walther et al., 2005), behavioral goals (Corbetta and Shulman, 2002; Ipata et al., 2006; Shomstein, 2012), and value-dependent appraisals (Phan et al., 2004; Pauli and Röder, 2008; Niu et al., 2012) on perceptual grouping. While a saliency map – in the context of bottom-up and top-down attention – has been proposed to exist in the intraparietal sulcus (Kusunoki et al., 2000; Goldberg et al., 2006), an interface between emotional and attentional processing could be attributed to regions of the value system and/or (value-based) ventral attentional system. Indeed, the neural correlates of emotional salience have been found in fMRI studies in amygdala, *insula*, *anterior cingulate* and *orbitofrontal cortex* (please note that our coarsely defined *OF* region combines both the orbitofrontal area itself as well as a large part of medial prefrontal cortex) (Phan et al., 2004; Chaudhry et al., 2009). The amygdala has been shown to be a crucial neural substrate for the processing of *primary inducers* which convey biologically relevant values (Davis and Whalen, 2001; Bechara et al., 2006). A separate neural circuit, comprising *orbitofrontal cortex* and *the anterior cingulate*, have been found to be responsible for the evaluation of *secondary inducers* conveying values based on an individual’s past experiences (Lane et al., 1997; Anderson et al., 2003). Notably, in some studies the right *insula* has been referred to as involving a fast, automatic processing of the *primary inducers* (Morris et al., 1998; Winston et al., 2002), while in others it has been attributed to evaluating the extent of personal emotional associations (Phan et al., 2004). In accordance with this dual role, recently a right anterior insula has been proposed as a key node for the experience-dependent integration of signals of salience across multiple sensory and semantic domains (Kleber et al., 2013, 2017).

The effects found in the early and intermediate phases of our experiments matches well with Adolphs’s scheme (Adolphs, 2002) that describes feedback influences assigned to the value system, both in terms of localization of regional responses and the sequence of regional activations. Indeed, the early effect in the extrastriate regions – that we ascribed to the repetition-based sensory learning (see section 4.2 of Discussion) – may alternatively be attributed to the change in the influence of *primary inducers*. These effects are usually detected at 90–120 ​ms after stimulus onset (Pizzagalli et al., 1999; Pourtois et al., 2004; Brosch et al., 2008) and are associated with elevated processing of stimuli carrying strong motivational cues. We, however, think this is a less likely explanation, because the disambiguation of Mooney figures did not (we assume) change biologically relevant aspects of low-level visual features and, moreover, the early effect was similar for face and tool stimuli – differing in the nature of their motivational salience. In any case, complex properties – extracted in the extrastriate regions – are in a position to feed into a network of value system regions; primarily *insula* and *orbitofrontal* cortex, in order to recognize *secondary inducers*. The exact timing of this activity is important as the original notion of a secondary inducer assumes that the episode recalling process – and its top-down influence – occurs rather late, at around 300 ​ms or later. However, in our experiment, the automatic and mandatory perception of *induced recognition* stimuli occurred with a rather short delay of about 70 ​ms, relative to the perception of *simply recognizable* images (see Table 1). This makes the involvement of declarative memory in this process unlikely. Right *insula* with its intermediate position in the treatment of primary and secondary inducers is well suited for the presentation of an experience-dependent perceptual prior that differs from episodic memory by being non-declarative (Ludmer et al., 2011; González-García et al., 2018).

We propose that this prior originates from a familiarity of an integrated saliency map which establishes new associative connections between conspicuous spatial features and value-dependent appraisals corresponding to a recognizable object. It has been shown that perirhinal cortex (which is included in our *entorh* region) supports familiarity-based recognition of novel associations, if the paired items are encoded as a single unit (Graf and Schacter, 1989; Haskins et al., 2008). Consistent with this “unitization hypothesis” behavioral studies suggest that encoding that promotes unitization of item pairs increases the familiarity of these novel associations (Quamme et al., 2007; Yonelinas et al., 1999). In parallel with this, it has been shown that: (1) medial temporal regions are involved in the effects of past experience on figure assignment (in the context of figure-ground segregation); and (2) competition for figural status can be biased by an agreement in a whole configuration familiarity and the part-level familiarity (Barense et al., 2012; Peterson et al., 2012). We suggest that training to recognize a Mooney figure by providing a whole configuration, in which the spatial features (not exactly matching the test image but having common configural properties) and value-based appraisals (of the recognizable object) are encoded as a single unit, results in a familiarity of integrated spatial and value-space saliency map. In this way, the extraction of appropriate spatial complex features from a visual stimulus provide partial cues about the unified configuration and promotes a reintegration of a whole configuration with a studied value-space part.

We interpret our effect in right *insula* as a manifestation of the formation of an integrated saliency map containing as a part a value-space configuration appropriate for appraisal of a face stimulus. The reintegration of the whole familiar saliency map based on partial cues, introduced by complex visual features (e.g. shapes), is accompanied by a transition from one value-space configuration to another and matches well to our finding that intermediate phase effect was associated with a change in the pattern of distributed neural activity, rather than simply an increase in power. We did not find a similar result for tool images in *FO* area, which we suggest is the result of worse signal-to-noise ratio of MEG recoding in this region. However, there was a pronounced similarity of RDM profiles for faces in *insula* and tools in *FO* that predicts that *FO* can perform the same reconfiguration of saliency map but for the processing of tools. The finding that new configurations allows better decoding between different examples of stimuli within-category (found for both face and tool related regions), can be attributed to the fact that in the new configurations, spatial configural features relevant for the categorization are strengthened (Jiang et al., 2007; Gillebert et al., 2009; Folstein et al., 2013). New integrated saliency maps for different samples within the group of recognized images include similar value-space parts, that separate them from unrecognizable stimuli, and different spatial parts corresponding to different visual configurations of degraded images that were relevant for the categorization. Thereby, an ability of activity to distinguish stimuli between unrecognizable and recognized images – at group level – emerged simultaneously with an increase of differentiation between samples within the group of recognized images only.

### 4.4 Semantic categorization for the *induced recognition* faces was delayed relative to *simply recognizable* faces but showed greater reliance on a value-dependent prior (“face-specificity”) than perception of *induced recognition* tools

Signals evident in the value system regions of the frontal lobe, in particular in the *insula*, may play a role in propagating some category-specific information that for example, reflect inferences about taste that are generated automatically when viewing pictures of food (Simmons et al., 2013; Martin, 2016). In the recent study (Leleu et al., 2019), it has been shown that a right-hemispheric neural signature of face categorization from natural images was significantly enhanced in the maternal vs. a control odor context in 4-month-old infants, providing strong support for value-dependent input driving categorization. However, the dimensionality of value-based space (Ekman, 1992; Adolphs, 2002; Fontaine et al., 2007) is likely too low for the number of possible configurations within it to cover the full spectrum of semantic categories. Moreover, a value-based configuration partially relates to the context-dependent appraisal of stimuli, rather than to a context-invariant semantic categorization. Rather than representing a category, this information is assumed to constrain a set of meaningful predictions making this set informative (narrowed) for a given stimulus. The selection of structural, context-invariant predictions in semantic space is related to the functions of the *FG* area and receives extracted cues from both extrastriate and value system regions (Vuilleumier et al., 2002; Adolphs, 2002; van de Riet et al., 2009).

It is reasonable to assume that the degree of narrowing of a set of predictions depends on the variety and proximity of a value-based experience with respect to a particular category formation. It has been previously shown that *simply recognizable* face perception relies more on activity in the right *FG* at the time window 140–180 ​ms (coinciding with a power-dependent N170 component), which is increased simultaneously to recognition facilitation and therefore is likely to rely more on value-dependent information (Kozunov et al., 2018). For *simply recognizable* tools, an additional action-based mechanism of left *IPS* was recruited later in time to complete a recognition event. In the present study, we have found that *induced recognition* faces were perceived without any effect in the N170 time window, which would distinguish them from processing of nonsense images (this however should not be interpreted as a complete absence of any processing). Only about 100 ​ms later than the time window of N170 component and, crucially, following the above effect in the *insula*, a processing of *induced recognition* faces revealed a partially power-dependent component that has been shown by temporal cross-category generalization analysis to be similar to the N170 component. On the one hand, this indicates that formation of a familiarity-based value priors for *induced recognition* faces require additional time and will delay semantic predictions compared to that when processing *simply recognizable* faces which is reflected in longer reaction times (see Table 1). On the other hand, even for *induced recognition* faces, the prior originating in the *insula* is informative enough to organize perception in value-dependent manner in the *FG*, whereas for the *induced recognition* tools this type of processing within the 240–290 ​ms window was not observed.

Unlike the completely power-dependent early effect, the face specific processing in the *FG* at 240–290 ​ms was determined by both an increase in power and a change in the pattern of neural activity. This may indicate that a value-dependent prior triggered in the *insula* acts to induce a subset of all the previous predictions in the *FG* (with higher precision). The more informative predicting variants are selected, the more modulatory gain from the value system they receive. This may explain both the appearance of a new activity pattern and a strengthening of the activity of the *rFG* region as a whole. In the recent RSA-based study (González-García et al., 2018), it was shown that the dimensionality of neural representational space increases towards higher-order brain areas and undergoes a particularly strong rise between extrastriate regions and *FG*. A complementary result was found for the preservation of neuronal patterns in these regions following disambiguation of Mooney images: pre-disambiguation patterns in extrastriate regions were mostly preserved following disambiguation and changed significantly in *FG*. This result agrees with our finding that in right *IO* and *MO* a disambiguation does not change activity patterns whilst in the *FG* it does. However, we have also shown this in a time-resolved manner for the transfer from the early phase of spatial configural features processing in *IO/MO* regions to the stage of semantic categorization. This transfer followed by an increase in dimensionality matches a key role of the *FG* in the representation of semantic categories combining influences of both visual and endogenous, value-dependent cues.

### 4.5 Parallel appraisal in the value system brain regions supplements predictive coding scheme with informative predictions on the top level

Priors in the prediction coding framework serve as constraints that reduce uncertainty when resolving the ill-posed inverse problem of perception (Pizlo, 2001; Friston and Kiebel, 2009). They provide agent-specific information required for the unambiguous recognition and propagate in top-down direction to modulate lower level sensory processing. To facilitate perception, predictions have to be available before recognition is accomplished. This requirement suggests a distinction between the origins of informative predictions in spatial and semantic spaces. Whilst spatial configural properties (such as those described by well-known Gestalt principles) are so persistent that they are believed to be innate, the meaning of an image can differ significantly from one individual to another and – with special relevance to this study – is experience-dependent. This suggests that low-level, visual space-based predictions are relatively fixed but informative because of their small number and innate nature. For high-level, semantic-space predictions to become informative, they must be pre-selected based on available visual cues. Our results allow us to supplement a simplistic predictive coding scheme and explain how informative predictions on higher levels arise from endogenous, value-dependent cues that contextualize lower-level processing.

This suggests a two-stage processing. At the first stage, a wide set of complex visual features are generated in parallel according to the predictive coding scheme, under the persistent constraints (i.e. priors) embodied by spatial configural (e.g., Gestalt) principles. A subset of these features serves as partial cues for re-activation of object-specific appraisal of visual input by the value system. The ensuing familiarity-based reintegration of the combined spatial and value-space saliency map contextualizes predictive coding across the hierarchy, integrating processing from striate/extrastriate regions through to the value system. The feedback from value systems to the high-level visual cortex, in particular*, the fusiform gyrus*, provides a fast selection of informative predictions and endows them with meaning. This influence was found more prominent for faces than for tools, which is in compliance with different dependence of these categories on value-related information. The scheme indicates that semantic predictions may emerge in higher order visual areas without category-specific inputs from lower order cortical visual areas, accounting for a non-hierarchical view of perception (Rossion et al., 2011). After a narrowed set of meaningful predictions is selected, the second stage of predictive coding proceeds; constructing a perceptually detailed representations of an object.

## CRediT authorship contribution statement

**Vladimir V. Kozunov:** Conceptualization, Methodology, Software, Formal analysis, Investigation, Writing - original draft, Writing - review & editing. **Timothy O. West:** Writing - review & editing. **Anastasia Y. Nikolaeva:** Investigation. **Tatiana A. Stroganova:** Supervision, Resources, Writing - review & editing. **Karl J. Friston:** Conceptualization, Resources, Writing - review & editing.
